# The Effect of a 7 Year-Long Cryopreservation on Stemness Features of Canine Adipose-Derived Mesenchymal Stem Cells (cAD-MSC)

**DOI:** 10.3390/ani11061755

**Published:** 2021-06-11

**Authors:** Santina Di Bella, Vincenza Cannella, Francesco Mira, Patrizia Di Marco, Antonio Lastra, Francesca Gucciardi, Giuseppa Purpari, Annalisa Guercio

**Affiliations:** Instituto Zooprofilattico Sperimentale della Sicilia “A. Mirri”, via G. Marinuzzi 3, 90129 Palermo, Italy; dottoremira@gmail.com (F.M.); patrizia.dimarco@izssicilia.it (P.D.M.); antonio.lastra@izssicilia.it (A.L.); francesca.gucciardi@izssicilia.it (F.G.); giuseppa.purpari@izssicilia.it (G.P.); annalisa.guercio@izssicilia.it (A.G.)

**Keywords:** canine adipose-derived mesenchymal stem cells (cAD-MSCs), cryopreservation, dimethyl sulfoxide (DMSO), fetal bovine serum (FBS)

## Abstract

**Simple Summary:**

The use of canine adipose tissue-derived mesenchymal stem cells represents a promising tool in the emerging field of autologous cell therapy in veterinary medicine. Cells need to be isolated and expanded in vitro in order to obtain the sufficient amount for clinical application, but long-term cultivation before therapeutic use is not recommended, since the cells may lose their stemness features. Alternatively, the cells can be cryopreserved and used as needed and in a short time after thawing. This study evaluated the effect of a 7 year-long cryopreservation using 10% dimethyl sulfoxide with different fetal bovine serum concentrations (from 10 to 90%) on different cell passages. The aim was to establish the most appropriate cell passage and serum percentage for the long-term cryopreservation of cells ensuring the maintenance of the stemness features. Cells were expanded in vitro from P0 to P1–P2 passages and subsequently frozen. This study demonstrated that a high percentage of serum (80%) is necessary to obtain optimal cryopreservation with 10% DMSO. Cells thawed at passages from P2 to P4, even after seven years, could be considered in the studies on therapeutic application and in the in vitro study, because they maintain stem potential after cryopreservation.

**Abstract:**

Mesenchymal stem cells (MSCs) are used in therapy in animal models and veterinary medicine, due to their capacity of inducing tissue regeneration and immunomodulation. Their clinical application requires a ready off-the-shelf amount of viable therapeutics doses. For this purpose, it is useful to cryopreserve MSCs to gain a ready and controlled source of abundant autologous stem cells. We evaluated the effect of 7 years cryopreservation using 10% dimethyl sulfoxide (DMSO) with different fetal bovine serum (FBS) concentrations (from 10 to 90%) on different passages of MSCs isolated from canine adipose tissue (cAD-MSCs). The study aimed to evaluate the most adequate cell passage and FBS percentage for the long-term cryopreservation of cells by maintaining the stemness features. Phenotype morphology, cell viability, osteogenic and adipogenic differentiation potentials, proliferative potential and expression of pluripotency markers were analyzed in thawed cells and compared with fresh ones. We demonstrated that cells cryopreserved with at least 80% FBS maintain unaltered the stemness characteristics of the freshly isolated cells. In particular, cells of P0–P1 passages have to be expanded in vitro and subsequently cryopreserved and cells of P2–P4 passages should be considered in the studies on therapeutic application and in vitro study of cAD-MSCs.

## 1. Introduction

Mesenchymal stem cells (MSCs) have attracted increasing attention due to their potential use in regenerative medicine and tissue engineering. These cells display a significant therapeutic plasticity as reflected by their advantageous characteristics: the ability to enhance tissue renovation, the immunomodulatory and anti-inflammatory effects [1]. Although MSCs can be easily isolated from several tissues, clinical use has favored adipose tissue because of its relative ease of stem cell recovery and the minimal donor-site morbidity [2]. In dogs, adipose tissue can be collected either by a simple adapted liposuction procedure, or through biopsies or in routine veterinary surgery procedures [3]. The visceral fat is particularly easy to collect because this species is subjected to a large number of ovariohysterectomies [4]. Interest in MSCs, both for regenerative and reparative therapies in dogs, is emerging as the current treatment options for several conditions that often do not result either in the desired clinical outcome or in the patients’ return to normal function [2]. Moreover, canine MSCs have been evaluated in some experimental and preclinical studies on efficacy and safety testing of novel treatments for humans, since the dog is considered to be a superior model for humans than rodents [2].

The clinical application of MSCs requires on demand access to a ready off-the-shelf amount of viable therapeutics doses. Cell dosage varies widely among applications and it is not established for any single treatment. MSCs require extensive culture expansion due to low cell number, and genetic alterations and contamination risks increase with culture time. For this purpose, it is very useful to cryopreserve these cells in order to gain a ready and controlled source of abundant autologous stem cells that maintain unaltered characteristics of the freshly isolated cells by preserving their vitality and maintaining their pluripotent phenotype. Cell aliquots can be biobanked for later administration immediately upon revitalization or after short-term expansion. Cryopreservation also increases MSC availability as frozen cells can be delivered over long distances [5].

Most cryopreservation protocols for stem cells use dimethyl sulfoxide (DMSO) at a concentration of 10% (*v*/*v*), often combined with fetal bovine serum (FBS) in concentrations ranging from 10% to 90% (*v*/*v*) [4,6,7,8]. FBS is commonly added to freezing solutions for its benefits to stabilize the cell membrane and adjust cell osmotic pressure [9]. On the other hand, it always accompanied with risk of infections due to its xenobiotic origin [10].

In this study, the effect of a 7 year-long cryopreservation using 10% DMSO with different FBS concentrations (from 10 to 90%) was evaluated on different cell passages of MSCs isolated from adipose tissue of *Canis familiaris* (cAD-MSCs). Phenotype morphology, cell viability, osteogenic and adipogenic differentiation potential, proliferative potential and expression of pluripotency markers were analyzed and compared in both fresh and thawed cells.

The aim was to establish the most appropriate cell passage and FBS percentage for the long-term cryopreservation of cAD-MSCs ensuring the maintenance of the stemness features.

## 2. Materials and Methods

### 2.1. Cell Culture

Samples were collected from visceral adipose tissue of 10 female dogs. Each sample was weighted, cleaned of large blood vessels and chopped, washed with Hank’s balanced salt solution (HBSS, Sigma–Aldrich^®^, Milan, Italy) and digested for 3–4 h at 37 °C with 0.2% collagenase type IA (GIBCO BRL/Life Technologies, Milan, Italy) prepared in sterile phosphate buffered saline (PBS) supplemented with 1% antibiotics (penicillin, streptomycin and amphotericin). The collagenase activity was neutralized by adding 10% FBS (EuroClone^®^, Milan, Italy). After centrifugation (300× *g* for 10 min) and washing of the pellet, cells were cultured in T25 flasks (Falcon, BD Bioscence, Basel, Switzerland), in non-inductive medium consisting of Dulbecco’s modified Eagle’s medium (D-MEM) low glucose (Sigma–Aldrich^®^, Milan, Italy) with 10% FBS and 1% penicillin, streptomycin and amphotericin. Cells were incubated at 37 °C in a humidified atmosphere containing 5% CO_2_. After the overnight incubation, non-adherent cells were removed and fresh medium was added to the flasks. The medium was renewed every 3 days. Adherent cells, grown to semiconfluency, were harvested, quantified and subcultured. A small volume of sterile and warm HBSS was added to the flasks for harvesting viable cAD-MSCs. HBSS was replaced with 500 µL of Trypsin/EDTA solution (0.5%) (Sigma–Aldrich^®^, Milan, Italy). Cells were resuspended in a culture medium, transferred from the flask to a sterile tube of 15 mL, and centrifuged at 300× *g* for 5 min. The supernatant was aspirated and the cells resuspended in a small volume of culture medium. Cells were counted using the hemocytometer (Cellometer Auto T4 EuroClone^®^, Milan, Italy). The primary cells cultured for 5–6 days were defined as passage ‘P0′. At 80% confluence, the MSCs were split and expanded (P1); cell expansion was continued until passage 6 (P6).

### 2.2. Cryopreservation and Thawing

At each passage from P1, cells were collected and resuspended at density of 1 × 10^6^ cells/cryovials in 1 mL of cryopreservation media composed of 10% DMSO (Sigma–Aldrich^®^, Milan, Italy) and increasing percentages (from 10% to 90%) of FBS. Cells were frozen by Mr. Frosty container (Thermo Fisher Scientific, Monza, Italy) decreasing −1 °C/min until −80 °C for 1 week and then they were transferred to liquid nitrogen tank for long time storage. After a 7 year-long cryopreservation, cells were thawed. cAD-MSCs were placed into a 37 °C water-bath for 1–2 min and washed in 90% D-MEM and 10% FBS to eliminate DMSO. Cells were counted and plated in T25 flasks with culture medium.

### 2.3. Cell Viability Analysis

Cryopreserved cAD-MSCs were thawed, the freezing solutions were removed and cell viability was assessed with a Trypan blue dye exclusion assay. A 1:2 dilution of the suspension was prepared using a 0.4% Trypan Blue solution (Sigma–Aldrich^®^, Milan, Italy). The number of the non-viable (stained) and viable (trypan blue excluded) cells were counted. Viability was expressed as the percentage of the number of the viable unstained cells obtained after thawing divided by the number of the viable cells before freezing.

The cell viability and proliferation rate for fresh and frozen-thawed cAD-MSCs were also evaluated by measuring 3-(4,5dimethylthiazol-2yl)-5-(3carboxymethoxyphenyl)-2-(4-sulfophenyl)-2H-tetrazolium inner salt (MTS), using a commercially available kit (Cell Titer 96 Aqueous One solution Cell proliferation Assay, Promega, Milan, Italy). Thawed cells were plated in a 96-well plate at a density of 1 × 10^4^ cells/100 µL per well. The Cell Titer 96 Aqueous One Solution Reagent (20 µL) was added and the plate was cultured for 4 h at 37 °C in a humidified 5% CO_2_ atmospheric environment. Plates were read on an absorbance microplate reader (Sunrise™, Tecan, Cernusco sul Naviglio (MI), Italy), complemented by universal reader control and data analysis software add-on (Magellan™, Tecan, Cernusco sul Naviglio (MI), Italy), at a wavelength of 492 nm.

### 2.4. Measurement of Cell Doubling Time and Cell Morphology Observation

The thawed cells were plated at a density of 2 × 10^4^ nucleated cells/cm^2^ in T25 culture flasks using the above-described culture medium. The medium was changed every 2–3 days until the adherent cell population reached 80% confluence. The adherent cAD-MSCs were passaged by digestion with Trypsin/EDTA solution (0.5%), counted with a hemocytometer, and a portion of the cells were reseeded for the subsequent passages (until P7) in T25 flasks (2 × 10^4^ cAD-MSCs/cm^2^). Cell-doubling time (DT) and cell-doubling number (CD) were calculated by hemocytometer counts and cell culture time (CT) for each passage according to the following 2 formulae:

CD = ln (Nf/Ni)/ln(2)(1)

DT= CT/CD(2)

where Nf is the final number of cells and Ni is the initial number of cells [11].

Cell-doubling time of fresh and cryopreserved cells (in medium with DMSO and 50% and 80% FBS) was compared.

Post-thaw cell morphology changes, such as cell enlargement, accumulation of vacuoles and presence of cellular debris were observed daily, at each passage, by inverted microscopy.

### 2.5. Differentiation Assay

To evaluate the stemness of established cultures, cells in P2, P4 and P6 after cryopreservation with 10% DMSO and 50% and 80% FBS were cultured in appropriate differentiation media and induced to differentiate toward adipogenic and osteogenic lineage.

#### 2.5.1. Adipogenic Differentiation

Cells were plated at 7500 cells/cm^2^ in 6-well plates and induced to differentiate in the StemMACS™AdipoDiff Media (Miltenyi Biotec, Bergisch Gladbach, Germany) differentiation medium for 21 days. Cells cultivated in basal medium (D-MEM, low glucose with 10% FBS) were used as a control. Differentiation and basal medium were changed every 48–72 h and cells were examined by inverted microscopy. The formation of lipid droplets was verified with Oil Red O staining.

#### 2.5.2. Osteogenic Differentiation

Cells were plated at 4500 cells/cm^2^ in 6-well plates and induced to differentiate in StemMACS™OsteoDiff Media (Miltenyi Biotec, Bergisch Gladbach, Germany) differentiation medium for 3 weeks. Cells cultivated in basal medium (D-MEM, low glucose with 10% FBS) were used as a control. Differentiation and basal medium were changed every 3 days and cells were examined by inverted microscopy. Calcified extracellular matrix deposits were detected by von Kossa staining.

### 2.6. Reverse Transcriptase-Polymerase Chain Reaction (RT-PCR)

Total RNAs were extracted from cells cryopreserved (in medium with DMSO and 50% and 80% FBS) using the RNAspin Mini RNA Isolation Kit (GE Healthcare, Milan, Italy) and treated with DNase I to remove contaminating DNA. cDNAs were synthesized from 1 µg total RNA using random hexamers and Superscript III reverse transcriptase (Thermo Fisher Scientific, Monza, Italy). Primers used for canine pluripotency markers (OCT4, NANOG and SOX2) were previously reported [12]. OCT4 and NANOG were amplified in 35 cycles at 94 °C for 1 min, 60 °C for 1 min, 72 °C for 1 min and followed by 72 °C for 5 min. SOX2 was amplified in 35 cycles at 94 °C for 1 min, 58 °C for 1 min, 72 °C for 1 min and followed by 72 °C for 5 min. PCR products were separated on 2% agarose gel by electrophoresis, stained with ethidium bromide and visualized under UV light.

### 2.7. Microbiological Control of cAD-MSCs and Reagents

cAD-MSCs were tested for possible contaminations during the steps of production. These quality controls included tests for bacteria and fungi, mycoplasma and viruses that were used as a part of routine and regular quality control screening procedures. To detect low level of contamination, samples from the cell cultures and reagents were inoculated onto solid media (blood agar, plate count agar and Sabouraud dextrose agar). PCR analysis were conducted for the screening of pestiviruses [13,14] and mycoplasma (MycoSensor PCR Assay Kit—Agilent Technologies, Santa Clara, CA, USA) that can contaminate cell cultures.

### 2.8. Statistical Analysis

The results are expressed as mean ± standard deviation. Data were normally distributed (*p* < 0.05, Kolmogorov–Smirnov test). Duncan’s multiple post-hoc comparison test was applied.

### 2.9. Ethical Statement

The study did not involve any animal experiment. Specimen collection was done 7 years earlier from dogs during routine spays required and authorized by dog owners and independent of this study. We had extracted adipose tissue samples from dogs only after their owners provided written informed consent. This study was conducted as part of the IZS SI 2007 RF research project entitled “Adult mesenchymal stem cells: differentiative lineages and applications in autologous and allogenic implantation and tissue remodelling”, approved by the Italian Ministry of Health.

## 3. Results

### 3.1. Culture, Expansion and Morphology of cAD-MSCs

**Figure 1:**
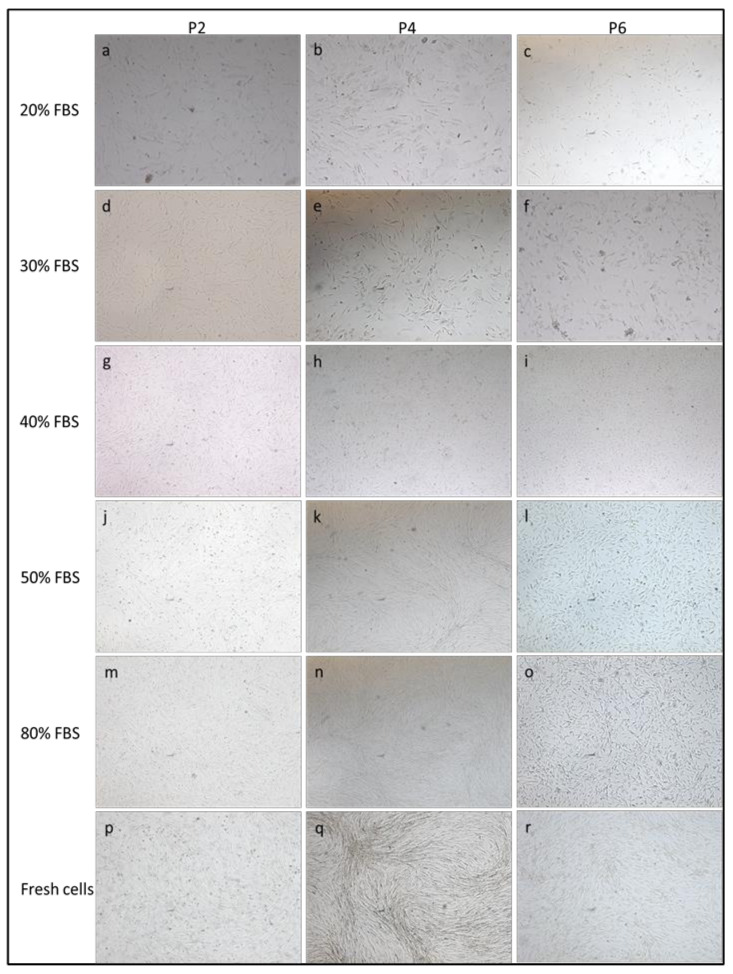
Morphology of cell cryopreserved with 10% DMSO—20%, 30%, 40%, 50% and 80% FBS percentages and fresh cells showed at passages P2 (**a**,**d**,**g**,**j**,**m**,**p**), P4 (**b**,**e**,**h**,**k**,**n**,**q**) and P6 (**c**,**f**,**i**,**l**,**o**,**r**). Cells frozen with FBS concentrations lower than 50%, after thawing, particularly in the passages P4–P6, showed an altered morphology, fusiform, polygonal, astroid shapes and a stunted growth in culture failing to reach semi-confluence. Cells from P2 to P6 passages, cryopreserved with serum percentages greater than 50%, maintained the ability to expand in culture and reached semiconfluence. Thawed cAD-MSCs presented fibroblast-like phenotype until passages P4 and demonstrated increased volume and cuboidal shape at P6 as in their fresh counterpart. A 100× magnification.

cAD-MSCs were isolated from canine adipose tissue and grown in plastic tissue culture flasks. At P0, adherent cells grew as spindle- or star-shape cells, forming colonies 3 days after plating. Cells became semiconfluent within 5–6 days. After passage P0, the cells began to proliferate rapidly and, as soon as they reached the semiconfluency (every 3–4 days), the cAD-MSCs were split and expanded until P6. After the first passage, MSCs adopted a fibroblast-like shape. Cell size and shape persisted until P5. Cells had increased volumes from passage P6. Moreover, cells cryopreserved at each passage in 9 cryopreservation media (composed of 10% DMSO and increasing percentages of FBS from 10% to 90%) were thawed after 7 years and retrospectively compared with fresh cells isolated from the same animals. Cryopreserved cells with serum percentages less than 50% showed stunted growth in the flask. Cells of all passages, cryopreserved with serum percentages greater than 50%, maintained the ability to expand in culture and reached semi-confluence. All thawed cAD-MSCs obtained from 10 dogs presented fibroblast-like phenotype until passages P4 and P5 and demonstrated increased volume and cuboidal shape at P6 as in their fresh counterpart (Figure 1). The cryopreservation of cells in liquid nitrogen in media containing DMSO with greater than 50% FBS percentages for 7 years and their thawing did not affect the cell morphology.

### 3.2. Cell Viability after Cryopreservation

**Figure 2:**
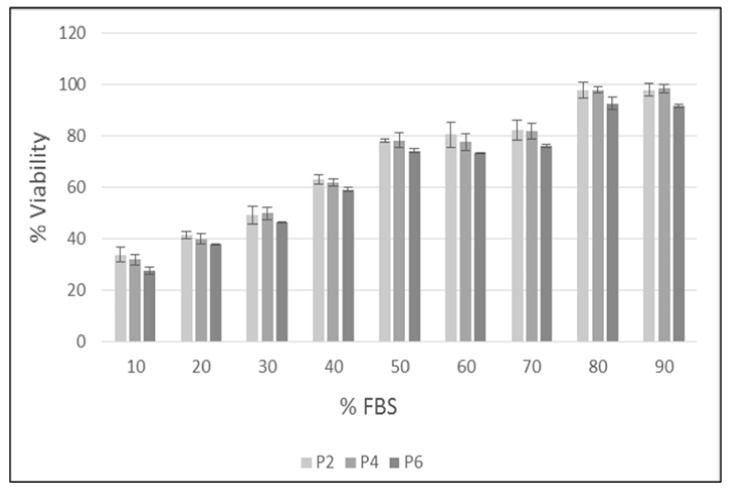
The effect of the different freezing solution composed of 10% DMSO and increasing percentage of FBS (from 10% to 90%) on post-thaw viability of cAD-MSCs at three passages (P2, P4 and P6).

The effects of different freezing solutions on post-thaw viability of cAD-MSCs at three passages (P2, P4 and P6) are shown in Figure 2. Cells cryopreserved with medium containing serum percentages up to 30% did not exceed 50% of cell viability after thawing at all cell passages. Frozen cells with 40% serum reached vitality values around 60%. cAD-MSCs cryopreserved at passages P2 and P4 with serum from 50% to 70% reached vitality percentages of about 80%. The viability of frozen cells with serum percentages of 80% and 90% reached 98% viability in passages P2 and P4. Cryopreserved cells at passage P6 with serum percentages from 10% to 90% showed lower viability percentages compared to passages P2 and P4.

### 3.3. cAD-MSCs Proliferation

**Figure 3:**
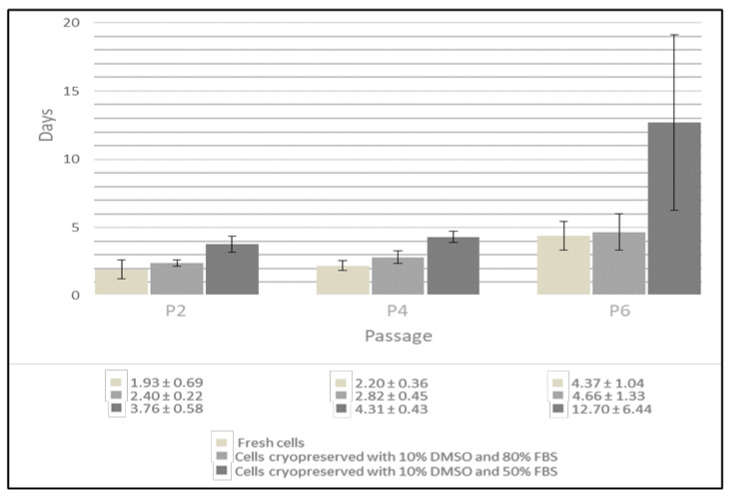
Analysis of population doubling time (pdt) of fresh cells and cells cryopreserved with 10% DMSO and 50% and 80% FBS (*n* = 10) at passages P2, P4 and P6. The pdt in fresh cells and in cells cryopreserved with 80% FBS shows the same trend in the three passages considered. Cells cryopreserved with 50% FBS show a delay in cell division at all passages compared to fresh cells.

To determine the proliferation potential, the population doubling time of cells cryopreserved with 10% DMSO and 50% and 80% FBS was calculated at passages 2, 4 and 6 and compared with that of the fresh cAD-MSCs (Figure 3). The analysis of proliferation capacity showed that doubling time is not significantly increased in passage P4 versus P2 in both fresh and cells cryopreserved with 10% DMSO and 80% FBS. However, the doubling time increased in P6. Cryopreservation with 10% DMSO and 50% FBS caused a delay of cell divisions at all passages.

### 3.4. Differentiation Potential

**Figure 4:**
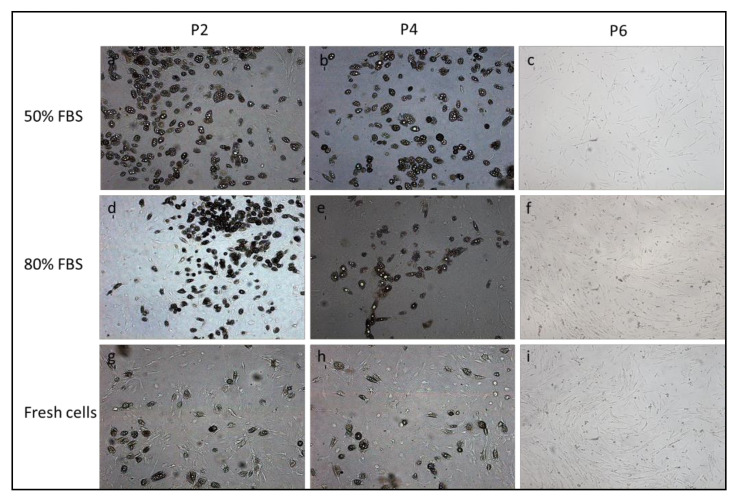
Fat globules appeared in cAD-MSCs, cryopreserved with 10% DMSO–50 and 80% FBS and thawed, cultured with adipogenic differentiation medium at passages P2 (**a**,**d**) and P4 (**b**,**e**). No differentiation was observed in the passage P6 (**c**,**f**). The frozen cells showed the same differentiation potential as fresh cells at each passage (**g**,**h**,**i**). A 100× magnification.

**Figure 5:**
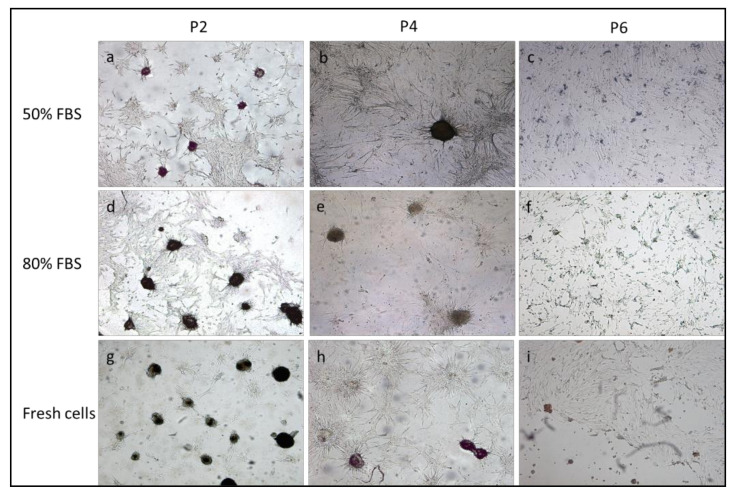
Deposition of calcified extracellular matrix revealed by von Kossa staining in cAD-MSCs, cryopreserved with 10% DMSO–50 and 80% FBS and thawed, cultured with osteogenic differentiation medium at passages P2 (**a**,**d**), P4 (**b**,**e**). The frozen cells showed the same differentiation potential as fresh cells at each passage (**g**,**h**,**i**). A 100× magnification.

Thawed cells of all dogs differentiated up to passage 4 across the two lineages tested. Differentiation was qualitatively assessed on the basis of cell morphology and histochemical stains. The cAD-MSCs induced toward adipogenic differentiation were analyzed for presence of intracellular lipid accumulation by Oil red O stain. MSCs in passages from P2 to P4 displayed many lipid droplets when compared with those in P6 passage (Figure 4). Thawed cells up to passage P4, induced toward osteogenic differentiation, formed aggregates and shown the mineralization of extracellular matrix marked by von Kossa staining (Figure 5). No differences were found in adipogenic and osteogenic potential between fresh and frozen cells.

### 3.5. Expression of Pluripotency Markers

**Figure 6:**
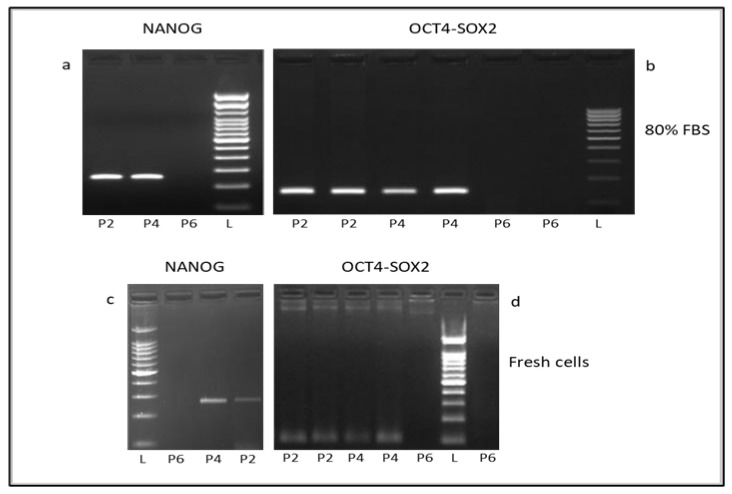
Representative images of expression of pluripotency-associated genes in cells cryopreserved with 10% DMSO and 80% FBS and thawed (**a**,**b**) and in fresh cells (**c**,**d**). NANOG (274 bp), OCT4 (141 bp) and SOX2 (142 bp) were expressed in passages P2 and P4 of cryopreserved and fresh cells. The gene expression was not revealed in P6.

A qualitative reverse-transcription PCR was used to assess the expression of the canine pluripotency associated transcription factors OCT4, SOX2 and NANOG in cell passages. These factors were not expressed in any post-thaw passage of cells cryopreserved with 10% DMSO and FBS concentration lower than 50% (data not shown). The pluripotency markers were maintained up to post-thaw passage P4 of cAD-MSCs cryopreserved with 10% DMSO and at least 50% FBS. The expression of OCT4, SOX2 and NANOG was not revealed in the P5 and P6 passages. The same results were previously observed in fresh cells (Figure 6) [12,15].

### 3.6. Microbiological Control of cAD-MSCs and Reagents

All the samples from the cell cultures examined were free from bacterial, viral and fungal contaminants.

## 4. Discussion

Adipose tissue is easily harvested with minimal risk to patients as compared to other stem cell sources. Stem cells derived from this tissue have been increasingly used for cell therapy both in humans and animals, either as freshly isolated or as cultivated AD-MSCs [15,16,17]. An important advantage of adipose-derived stem cells is their abundance: from 1 g of adipose tissue an average of 0.5–2.0 × 10^6^ stromal vascular fraction cells can be isolated, which gives 1–10% of stem cell yield [18]. These cells proliferate rapidly with high cellular activity and have a great potential of differentiation into multilineage cells, making them an ideal source to obtain MSCs [19]. For autologous use, the adipose tissue is collected 2 or 3 weeks before the treatment and the animal receive the cultivated cells, but long-term cultivation before therapeutic use is not recommended, since the cells may lose their progenitor characteristics [20]. Alternatively, the cells can be isolated, expanded and subjected to long-term storage, so that they can be used as needed and in a short time after thawing. Cryopreservation and biobanking allows for MSC to be prepared in large batches, under the application of accepted quality control measures to ensure their safety. To assure that cryopreservation medium does not alter the stemness characteristics and the differentiation potential of isolated MSC is also of primary importance [21]. Advances in cell therapy, stem cell research, personalized medicine and cell banking drive the need for optimize storage protocols.

To address this issue, the study was conducted to determine the effect of long-term cryopreservation on different passage of cAD-MSCs cryopreserved with ten different media composed of 10% DMSO and ten different concentrations of serum. DMSO is standard cryoprotectant used to stabilize cell protein and membrane and to prevent intracellular ice formation [22,23]. FBS stabilizes the cell membrane and adjust cell osmotic pressure [9]. Data obtained demonstrated that cells cryopreserved at the study condition with high FBS percentages show similar stem characteristics as fresh ones.

Viability is the primary indicator on cryopreservation success and its >70% value for cryopreserved cells is generally considered a post-thaw viability threshold generally accepted and the benchmark for clinical application [24]. This study demonstrated that cAD-MSCs up to passage P4 cryopreserved with 50% FBS showed >80% viability. This reached almost 100% in cells with 80–90% FBS. Cells with more than 50% FBS, cryopreserved in liquid nitrogen and thawed after 7 years showed similar morphological characteristics and proliferative ability as fresh cells. cAD-MSCs grew within a monolayer adhering to the culture flask bottom with fibroblast-like shape [25] until passages P4. Cell morphology gradually became diversified with increasing passages, accompanied by an increase in cell size changes as in their fresh counterpart [12,26]. The proliferation kinetics of cAD-MSCs from passages P1 to P7 were examined in cells cryopreserved with 10% DMSO and 50 and 80% FBS. While there were no significant differences in the population doubling time from passage P2 to P4, passage P6 displayed a longer doubling time. Moreover, doubling time increased at all passages for the cells cryopreserved with 50% FBS with respect to fresh cells.

The expression of pluripotent markers OCT-4, NANOG and SOX-2 was revealed up to passage P4 of cells cryopreserve with more than 50% FBS and not at P5 and P6 passages. These pluripotent transcription factors regulate the self-renewal and differentiation abilities of AD-MSCs [27]. OCT4 and NANOG are not only essential for the maintenance of pluripotency in embryonic stem cells but also in maintaining MSC properties. SOX2 is also important for maintaining proliferation and osteogenic differentiation potential of MSCs. OCT4 and SOX2 are usually expressed at low levels in early-passage MSCs and gradually decrease as the passage number increases [28]. The use of a quantitative RT-PCR in this study would have probably allowed to better appreciate this aspect.

The success of utilizing stem cells in tissue-engineering applications is highly dependent on maintaining a satisfactory level of differentiation potential after extensive in vitro expansion [26]. In addition, in agreement with other studies [4,29], differentiation was not affected by the cryopreservation process. Thawed cells were able to differentiate into two mesodermal lineages. Adipogenic differentiation was accompanied by cell’s shape change from a fibroblast to a large rounded morphology and by accumulation of small and non-uniform lipid droplet. Osteogenic induction medium caused the formation of cell aggregates and matrix mineralization that was assessed by calcium specific von Kossa staining. Moreover, it was assessed that cell cultures were free from microbiological contamination. Specific test for the detection of bacteria, yeast, fungi, mycoplasma and viruses should be used as a part of routine and regular quality control screening procedures. Mycoplasma competes with the cells for the nutrients in the culture medium, typical signs of contamination consist in the reduction of the rate of cell proliferation, and changes in cellular physiology including gene expression, metabolism and phenotype. Biosafety assessment of cryopreserved MSCs is necessary to ensure the safe use of cells prior to clinical application. Frozen cells may have the advantage over fresh cells of being more controlled for the presence of contaminants that may come from the animal of origin or from handling. Further studies will have to be conducted to evaluate the use of cryoprotectants that allow the reduction of FBS concentrations with a view to reducing xenocontaminants.

## 5. Conclusions

This study demonstrated that a high percentage of FBS (at least 80%) is necessary to obtain optimal cryopreservation of cAD-MSCs with 10% DMSO. To provide the needed doses for clinical studies, cells from lower passages (P0, P1) have to be expanded in vitro and subsequently frozen for storage/banking purposes. Probably, a short recovery period post-thaw in culture may facilitate a regain of function [30]. Our data suggest that, cells thawed at from passages P2 to P4, even after seven years, could be considered in the studies on therapeutic application as well as in vitro study of cAD-MSCs, because they maintain their stem potential after cryopreservation.

## Data Availability

Data are contained within the article.
